# Histone H4 Lysine 12 Lactylation Promotes the Senescence of Alveolar Epithelial Type II Cells in Chronic Obstructive Pulmonary Disease by Modulating the CD38-NAD^+^ Signaling Pathway

**DOI:** 10.14336/AD.2025.0291

**Published:** 2026-10-16

**Authors:** Chunxiao Yang, Qi Wang, Yuhan Xiong, Nan Ruan, Jun Yu, Yi Huang, Yu Liu, Cuntai Zhang, Weiwei Yu

**Affiliations:** ^1^Department of Geriatrics, Institute of Gerontology, Tongji Hospital of Tongji Medical College, Huazhong University of Science and Technology, Wuhan, China.; ^2^Key Laboratory of Vascular Aging, Ministry of Education, Tongji Hospital of Tongji Medical College, Huazhong University of Science and Technology, Wuhan, China.; ^3^Department of Cardiothoracic Surgery, Institute of Gerontology, Tongji Hospital of Tongji Medical College, Huazhong University of Science and Technology, Wuhan, China.

**Keywords:** Epigenesis, Genetic, DNA Methylation Aging, Biological Clocks, Biomarkers

## Abstract

In chronic obstructive pulmonary disease, the senescence of type II alveolar epithelial cells is a key driver of disease progression, severely impacting lung function and structure. Lactate accumulation, a common feature of chronic hypoxic conditions such as COPD, is increasingly recognized for its role in modulating cellular functions via epigenetic mechanisms. This study aimed to investigate the specific effects of lactate-induced histone lactylation on AEC2 senescence and its contribution to COPD progression. Our experiments revealed a significant increase in histone lactylation levels in COPD models, with site-specific screening identifying histone H4 lysine 12 lactylation as a predominant modification. Using the Cleavage Under Targets and Tagmentation technique (CUT&Tag) sequencing, we demonstrated that H4K12la modulates the CD38-nicotinamide adenine dinucleotide (NAD^+^) signaling pathway, thereby promoting AEC2 senescence and exacerbating COPD progression. Further *in vitro* and *in vivo* analyses confirmed that elevated H4K12la expression was associated with increased CD38 levels and decreased NAD^+^ concentrations. To interrogate this pathway, we employed the p300/CBP inhibitor A485, which specifically inhibits H4K12la levels. This intervention significantly improved AEC2 senescence and reduced COPD-related pathology. Subsequently, we explored additional therapeutic strategies using the CD38 inhibitor 78c and the NAD^+^ precursor β-nicotinamide mononucleotide (NMN), both of which effectively reduced senescence markers and further ameliorated COPD symptoms. These findings highlight the critical role of lactate-induced histone lactylation, specifically H4K12la, in COPD pathogenesis. Targeting the H4K12la-CD38-NAD^+^ axis, with strategies such as p300/CBP inhibition, offers promising therapeutic avenues for managing the disease.

## INTRODUCTION

Chronic obstructive pulmonary disease (COPD) is a significant global health concern characterized by persistent respiratory symptoms and irreversible airflow limitation [1]. A critical aspect of COPD pathogenesis is the accelerated senescence of type II alveolar epithelial cells (AEC2s), which are essential for maintaining alveolar integrity, surfactant production, and repair following lung injury [2, 3]. Cigarette smoke (CS) exposure - the leading cause of COPD - induces oxidative stress and chronic inflammation in the lungs [4]. This oxidative stress results in mitochondrial dysfunction and iron dysregulation within AEC2s, promoting a pro-senescent phenotype characterized by impaired energy metabolism and increased production of reactive oxygen species [5, 6]. Iron dysregulation further exacerbates oxidative damage through the generation of harmful radicals [7]. Consequently, these cellular changes diminish the regenerative capacity of AEC2s and increase the release of pro-inflammatory mediators, exacerbating lung injury and fibrosis. Furthermore, chronic inflammation disrupts Wnt signaling pathways, leading to the depletion of lung progenitor cells and further compromising tissue regeneration [8]. Therefore, targeting senescent AEC2s represents a potential therapeutic strategy for COPD. Emerging evidence suggests that lactate accumulation, a common consequence of hypoxia and inflammation observed in COPD, may further drive AEC2 senescence [9]. In patients with COPD, impaired airflow and gas exchange lead to hypoxic conditions within the lung microenvironment, prompting cells to shift towards anaerobic glycolysis that results in increased lactate production [10]. Lactate functions as a signaling molecule that induces histone lactylation, an epigenetic modification in which lactate moieties are added to lysine residues on histone tails, altering chromatin structure and gene transcription [11]. Zhang et al. (2019) demonstrated that histone lactylation can regulate gene expression profiles associated with inflammation and metabolism [12]. In AEC2s, elevated lactate levels may induce histone lactylation at promoters of senescence-associated genes, such as cyclin-dependent kinase inhibitor 1A (p21), tumor protein 53 (p53), cyclin-dependent kinase inhibitor 2A (p16) and phosphorylated histone H2AX (γ-H2AX), leading to cell cycle arrest and reduced regenerative capacity [13]. This impairment exacerbates alveolar damage and contributes to disease progression in COPD. While histone lactylation has been linked to various cellular processes, its specific impact on AEC2 dysfunction in COPD remains unclear. Understanding this pathway is critical for identifying novel therapeutic targets aimed at restoring AEC2 function and halting disease progression.

CD38 is a multifunctional enzyme that hydrolyzes nicotinamide adenine dinucleotide (NAD^+^) to produce cyclic ADP-ribose (cADPR), a potent second messenger that mobilizes calcium from intracellular stores [14]. By regulating NAD+ levels and calcium signaling, CD38 plays a critical role in cellular energy metabolism, DNA repair, stress resistance, and longevity [15, 16]. NAD^+^ is essential for the activity of sirtuins, a family of NAD^+^-dependent deacetylases that promote genomic stability and mitochondrial function; thus, NAD^+^ depletion is closely linked to cellular senescence [17]. Moreover, CD38-mediated production of cADPR alters calcium-dependent signaling pathways, activating transcription factors such as nuclear factor kappa-B (NF-κB) and nuclear factors of activated T cells (NFAT) that drive the expression of pro-inflammatory cytokines [18]. In COPD, upregulation of CD38 in airway smooth muscle reduces NAD^+^ availability and disrupts calcium homeostasis, promoting cellular senescence and exacerbating inflammatory responses within the lung tissue [19]. Despite these findings, the upstream mechanisms regulating CD38 expression, particularly the influence of epigenetic modifications like histone lactylation, remain largely unexplored. Understanding how these epigenetic factors modulate CD38 may lead to targeted therapies to restore NAD^+^ levels, normalize calcium signaling, and mitigate disease progression.

Chronic hypoxia-induced lactate accumulation in COPD is hypothesized to drive alveolar epithelial dysfunction through epigenetic remodeling. While histone lactylation has emerged as a hypoxia-sensitive post-translational modification, its specific role in regulating AEC2 senescence and the underlying mechanisms remain unexplored. In this study, we aim to characterize the spatial distribution of histone lactylation in COPD-affected AEC2s, identify the key lactylation sites modulating NAD^+^ metabolism and cellular senescence, and evaluate therapeutic strategies targeting the lactate-epigenetic-aging axis to mitigate COPD progression.

## MATERIALS AND METHODS

### Cell Culture and Treatment

A549 cell line was obtained from the Shanghai Institute of Cell Biology, Chinese Academy of Sciences (Shanghai, China). Primary mouse AEC2s were isolated as previously described [10]. All cells were maintained in Dulbecco's Modified Eagle Medium (DMEM) supplemented with 10% fetal bovine serum, 100 U/mL penicillin, and 100 mg/mL streptomycin, and incubated at 37 °C in 5% CO_2_. Cells were passaged at a 1:3 ratio every two days. For the LDHA treatment group, A549 cells were treated with 10 μM GNE-140 for 24 hours after cigarette smoke extract (CSE) stimulation [20]. For the A485 treatment group, A549 cells were treated with 10μM A485 for 48 hours after CSE stimulation. For the 78c treatment group, A549 cells were pretreated with 1.0 μM 78c for 4 hours before CSE stimulation [21]. For the NMN treatment group, A549 cells were pretreated with 100μM NMN for 4 hours before CSE stimulation [22].

### Animal Model and Treatment

C57BL/6 mice (aged 8-10 weeks) with a model of CS-induced COPD were procured from the Wuhan Zhongke Experimental Animal Co., Ltd. in Wuhan, China. These mice were maintained in sterile environments, adhering to a strict 12-h light/dark cycle, and were given 1 week for acclimatization. The CS-induced COPD model used was modified from He et al [23]. Briefly, mice were exposed to smoking from 3R4F cigarettes (Kentucky University) twice a day (24 cigarettes per day), 5 days per week for 12 weeks. Pulmonary function was assessed by a forced pulmonary maneuver system (Buxco Research Systems, Wilmington, USA), and paraffin sections of the lung tissues were obtained from every mouse to confirm the COPD phenotypes. COPD mice received CD38 inhibitor 78c dissolved in 5% dimethyl sulfoxide (DMSO; 15 mg/kg/day) [24] and NMN dissolved in 5% phosphate buffer saline (PBS; 300 mg/kg/day) via oral gavage starting from week 8 of CS exposure and continuing for 4 weeks [25]. The p300/CBP inhibitor A485 dissolved in 5% DMSO (25 mg/kg/day) was administered via oral gavage to COPD mice. The administration was performed daily for 4 weeks [26]. Mice were monitored post-gavage to ensure no adverse effects. Control mice were housed under identical conditions but exposed only to ambient air. All experimental animal procedures were approved by the Tongji Medical College of HUST University Committee on Animal Care and adhered to the National Institutes of Health Guidelines on the Use of Laboratory Animals.

### Human Lung Sample

Human lung tissue for immunohistochemical staining was derived from three patients with COPD, and three non-COPD, cancer-free controls were collected at Tongji Hospital. Written informed consent was obtained from all participants. A COPD diagnosis was made according to consensus diagnostic criteria from the Global Strategy for the Prevention, Diagnosis, and Management of COPD: 2024 Report (https://goldcopd.org/2024-gold-report/). The entire study was conducted in compliance with the Declaration of Helsinki and approved by the Human Assurance Committee of Tongji Hospital. COPD Inclusion Criteria: (1) Diagnosed with COPD per GOLD 2024 (post-bronchodilator FEV_1_/FVC < 0.70). (2) Age 40-80 years with ≥10 pack-years of smoking (current or former). (3) Clinically stable (no exacerbations requiring hospitalization or systemic corticosteroids in the past 4 weeks). (4) No history of asthma or other chronic respiratory diseases (e.g., interstitial lung disease). COPD Exclusion Criteria: (1) History of asthma or other chronic respiratory diseases (e.g., bronchiectasis, cystic fibrosis). (2) Severe cardiovascular, hepatic, or renal dysfunction (e.g., NYHA III/IV heart failure, eGFR < 30 mL/min/1.73 m²). (3) Active malignancy (within 5 years, except non-melanoma skin cancer). (4) Recent acute respiratory infection (within 4 weeks). (5) Use of immunosuppressive or corticosteroid therapy (≥10mg/day prednisone equivalent for ≥4 weeks). Healthy Control Definition:(1) No history of COPD, asthma, or other chronic respiratory diseases. (2) Post-bronchodilator FEV_1_/FVC ≥ 0.70. (3) No history of lung cancer or malignancies (except non-melanoma skin cancer). (4) Non-smokers or ex-smokers with <10 pack-years and quit ≥10 years ago. (5) Age-, sex-, and smoking status-matched to COPD patients. (6) No chronic medical conditions requiring treatment (e.g., diabetes, hypertension). Clinical characteristics of patients (age, gender, smoking history, FEV_1_/FVC) are now summarized in Table 1.

**Table 1:** Clinical data of subjects.

	Control1	Control2	Control3	COPD1	COPD2	COPD3
**Age (years)**	50	53	68	66	53	70
**Sex**	Male	Female	Male	Male	Male	Male
**Smoking history (years)**	30	0	0	40	30	25
**Daily number of cigarettes**	2	0	0	40	20	20
**FEV1/FVC**	80.05	77.01	79.89	54.56	53.26	52.49
**FEV1% predicted**	111.6	113.7	98.4	50	57.1	85.7
**Acute exacerbations per year**	0	0	0	3	2	1
**mMRC score**	0	0	0	2	2	1
**CAT score**	0	0	0	22	18	13

FVC: Forced vital capacity. FEV1: Forced expiratory volume in one second

### Preparation of Cigarette Smoke Extract (CSE)

CSE was prepared as previously described [23]. Smoke from four cigarettes containing 10 mg tar, 0.9 mg nicotine, and 12 mg CO was vaporized into a flask containing 20 mL DMEM (having a temperature of 37 °C) using a vacuum pump at a constant speed. Each cigarette was smoked for 5 minutes. The pH of the resulting solution was adjusted to 7.4, filtered through a 0.22-μm pore filter, and considered to be 100% CSE. For quality control, CSE quality was accepted if the Δoptical density at 540 nm was less than 0.1.

### Antibodies

P21 monoclonal antibody (sc6246, Santa Cruz, USA), P21 monoclonal antibody (ab109520,Abcam,UK), P53 monoclonal antibody (ab26,Abcam, UK), P16 monoclonal antibody (ab61243, Abcam, UK), γ-H2AX monoclonal antibody (ab81299, Abcam, UK), GAPDH polyclonal antibody (10494-1-AP, Proteintech, China), SP-C monoclonal antibody (ab211326, Abcam, UK), DAPI (ab228549, Abcam, UK), l-Lactyl Lysine monoclonal antibody (PTM-1401RM, PTM BIO, China), H4K8la monoclonal antibody (PTM-1415RM, PTM BIO, China), H3K23la monoclonal antibody (PTM-1413RM, PTM BIO, China), H4K12la monoclonal antibody (PTM-1411RM, PTM BIO, China), H3K18la monoclonal antibody (PTM-1406RM, PTM BIO, China), H4 monoclonal antibody (PTM-685RM, PTM BIO, China), CD38 polyclonal antibody (60006-1-Ig, Proteintech, China), CD38 monoclonal antibody (AB108403, Abcam,UK), F4/80 polyclonal antibody (70076, Cell Signaling Technology, USA), Alexa Fluor 488-conjugated goat anti-rabbit IgG (715-545-150, Jackson ImmunoResearch, China), Alexa Fluor 555-conjugated goat anti-mouse IgG (115-565-071, Jackson Immuno Research, China).

### siRNA Transduction

RiboBio genOFF™ siRNAs against mouse CD38 were purchased from Guangzhou RiboBio Co., Ltd. (Guangzhou, China). A549 cells were seeded into 6-well plates to transfect with siRNA using Lipofectamine 2000 for 6 hours. Then the medium was replaced. After transduction for 48 hours, A549 cells were treated with OA/PA for 24 hours. The efficiency of gene knockdown was evaluated by western blotting and real-time polymerase chain reaction (PCR).

### Real-Time Reverse Transcription Polymerase Chain Reaction (RT-qPCR)

Total RNA was extracted using Buffer RL reagent (Vazyme Biotech), and 1 μg RNA was reverse transcribed into cDNA using HiScript II Q Select RT Supermix. Amplification was performed using Power SYBR Green Master Mix and a LightCycler 96 instrument (Roche, Switzerland). The 2-ΔΔCT method was used for quantification. Primer sequences are as follows: CD38-F: 5'-GCTGTGAGTGATGCTGCTGA-3'; CD38-R: 5'-CTG GGCTTGGTGTTGTTGTC-3'. CaSR-F: 5'-AGGAGAG GCTGCTTTGACTG-3'; CaSR-R: 5'-GCTGAGGAGCA GGTTGAGTT-3'. NFAT-F: 5'-ATGGCAGAGACAGT GACGGA-3'; NFAT-R: 5'-CCTGAGGAGGGAGGTTG ACT-3'. β-actin-F: 5'-CATGTACGTTGCTATCCAGG C-3'; β-actin-R: 5'-CTCCTTAATGTCACGCACGAT-3'.

### Enzyme-linked immunosorbent assay

According to the manufacturer’s recommendations, we used ELISA kits (Neobioscience, China) to measure the concentrations of interleukin-6 (IL-6), chemokine ligand 2 (CCL2), chemokine ligand 5 (CCL5), and chemokine (C-X-C motif) ligand 1 (CXCL1) in the supernatants of alveolar epithelial cell culture. The absorbance (OD value) was determined with a microplate reader at a wavelength of 450 nm. The concentrations of IL-6, CCL2, CCL5, and CXCL1 in cell supernatants were calculated from a standard curve.

### Histological Analysis

Lung tissues were fixed, dehydrated, embedded in paraffin, and sectioned into 4-μm-thick slices using a microtome. Hematoxylin and eosin (H&E) and Masson staining were performed. Emphysematous changes were assessed by mean linear intercept (MLI), mean alveolar septal thickness (MAST), and destructive index (DI). The measurement of the MLI was carried out according to the method of Jiansheng Gao et al: A “cross” line was drawn in the center of the visual field, then the number of alveolar septa (Ns) passing through this cross line was counted, and the total length (L) of the cross line was measured. Finally, the average lining spacing is calculated by the formula MLI = L/Ns. The value of MLI can directly reflect the average diameter (μm) of alveoli [27, 28]. The determination of the alveolar DI was carried out in accordance with the method of Chen Yan et al. Three H&E-stained sections were taken from each mouse. At least 20 non-repetitive fields of view were observed under the low-power microscope for each section. The destruction of alveolar structure was recorded as normal (N) or destroyed (D). The sum of N and D values of each section in each case was required to be greater than 3000. DI was calculated by the formula: D/(D+N) × 100 [29]. MAST refers to the average thickness of the alveolar septum (alveolar wall), that is, the average width of the tissue that separates adjacent alveoli. Three H&E-stained sections were taken from each mouse. For each section, multiple alveolar intervals were randomly selected under a high-power microscope, their thicknesses were measured, and the average values were calculated.

### Immunofluorescence Staining

Mouse lung tissues were harvested, fixed in 4% paraformaldehyde at 4 °C overnight, and dehydrated in a graded series of sucrose solutions. The tissues were embedded in OCT compound and cryosectioned at a thickness of 5-10 µm using a cryostat (Leica CM3050S). Tissue sections were mounted on glass slides and air-dried at room temperature for 30 minutes. For immuno-fluorescence staining, the sections were rehydrated with PBS and permeabilized with 0.3% Triton X-100 in PBS for 10min. After permeabilization, tissue sections were blocked with 5% bovine serum albumin (BSA) in PBS for 1h at room temperature to prevent non-specific binding. The sections were then incubated with primary antibodies, diluted in PBS with 1% BSA, overnight at 4 °C. Following primary antibody incubation, the sections were washed three times with PBS for 1 hour at room temperature in the dark. Next, the membrane was incubated with an HRP-conjugated secondary antibody. For nuclear counterstaining, DAPI (1 µg/mL) was added for 5min. After staining, the sections were washed three times in PBS, mounted with anti-fade mounting medium (Beyotime P0098), and covered with a coverslip. Isotype antibody controls and secondary antibody only controls were used to demonstrate the specificity of the antibodies and the specific interaction between the primary antibody and its target antigen. Confocal images were acquired using a laser-scanning confocal microscope (Leica TCS SP8) with 40× or 63× oil immersion objectives. Image analysis was performed using ImageJ software (NIH), and the colocalization of H4K12la, p16, γ-H2AX, CD38, or p21 with AEC2-positive cells or CD38 with macrophage-positive cells was quantified using the colocalization plugin in ImageJ (Nikon).

### Protein Extraction and Western Blotting

Tissues or cells were lysed with RIPA lysis buffer containing protease and phosphatase inhibitors for 30 minutes on ice, centrifuged at 12,000 rpm for 15 minutes at 4 °C, and the supernatants were collected. The protein concentration was determined using the BCA method, and the final concentration was adjusted to ensure consistency. The protein samples were mixed with protein loading buffer and boiled at 98 °C for 10 minutes. Next, 30 μg of protein was separated by 12% sodium dodecyl sulfate-polyacrylamide gel electrophoresis (SDS-PAGE). After electrophoresis, the proteins were transferred onto polyvinylidene difluoride membranes (0.45-μM pore size). The membranes were blocked with TBST buffer containing 5% skim milk powder for 2 hours at room temperature, then incubated with the corresponding primary antibodies overnight at 4 °C on the shaker. Membranes were washed three times with TBST buffer and then conjugated with a horseradish peroxidase-labeled secondary antibody for 2 hours at room temperature. Protein bands were visualized using the ChemiDoc Acquisition Image XRS + system (Bio-Rad Laboratories). Protein expression levels were semi-quantified using ImageJ software (version 1.52). GAPDH was used as the loading control.

### Senescence-Associated β-Galactosidase (SA-β-gal) Activity

SA-β-gal activity was performed as reported previously [30]. Briefly, cells were washed once with PBS and fixed with β-galactosidase staining solution for 15 minutes at room temperature. Cells were washed with PBS three times for 3 minutes each. Cells were treated with staining solution (prepared according to the C0602 instructions) and sealed with a sealing film. After overnight incubation at 37 °C, the cells were imaged using an Olympus IX53 + DP73 inverted microscope.

### Cleavage Under Targets and Tagmentation (CUT&Tag)

CUT&Tag was performed with the Hyperactive In-Situ ChIP Library Prep Kit for Illumina (pG-Tn5) (TD901; Vazyme Biotech) according to the manufacturer’s instructions. In brief, AEC2 cells were collected and bound to Concanavalin A-coated beads. Subsequently, cells were resuspended in antibody buffer and incubated with primary antibodies against H4K12la and then with secondary antibodies in sequence. The samples were incubated with pA-Tn5 transposase. After transposon activation and tagmentation, DNA was isolated, amplified, and purified to construct a sequencing library. The library for sequencing was constructed, and VAHTS DNA Clean Beads (N411; Vazyme Biotech) were used for the purification steps. The library was quantified with the VAHTS Library Quantification Kit for Illumina (Vazyme Biotech) and sequenced on an Illumina NovaSeq 150PE. CUT&Tag technology offers superior sensitivity and requires fewer cells (<10,000 cells per assay), making it particularly suitable for studying rare cell populations such as primary alveolar epithelial cells in clinical samples. Additionally, CUT&Tag minimizes background noise through in situ chromatin targeting and Tn5 transposase-based tagmentation, enabling precise identification of histone modification sites even under low-abundance conditions. These technical advantages position CUT&Tag as an ideal tool to dissect the spatial dynamics of lactate-induced histone lactylation in COPD pathogenesis.

### Chromatin Immunoprecipitation Assay (ChIP)

Isolated AEC2 cells were crosslinked with 1% formaldehyde for 10 minutes at room temperature and quenched by the addition of glycine to a final concentration of 125 mM for 5 minutes. The fixed cells were resuspended in lysis buffer (1% SDS, 5 mM EDTA, and 50 mM Tris-HCl, pH 8.1) containing protease inhibitors, then subjected to 30 cycles (30 s on and 30 s off) of sonication (Bioruptor, Diagenode) to generate chromatin fragments of approximately 300 bp in length. Lysates were diluted in buffer (1% Triton X-100, 2 mM EDTA, 150 mM NaCl, and 20 mM Tris-HCl, pH 8.1) containing protease inhibitors. For immunoprecipitation, the diluted chromatin was incubated with normal IgG (control), H4K12la, or H3K18la antibodies overnight at 4 °C with constant rotation, followed by incubation with 50 μL of 50% (v/v) protein A/G Sepharose beads for an additional 2 hours. Beads were successively washed with the following buffers: TSE I (0.1% SDS, 1% Triton X-100, 2 mM EDTA, 150 mM NaCl, and 20 mM Tris-HCl, pH 8.0); TSE II (0.1% SDS, 1% Triton X-100, 2 mM EDTA, 500 mM NaCl, and 20 mM Tris-HCl, pH 8.0); TSE III (0.25 M LiCl, 1% Nonidet P-40, 1% sodium deoxycholate, 1 mM EDTA, and 10 mM Tris-HCl, pH 8.0). The pulled-down chromatin complex was eluted by TE (1 mM EDTA and 10 mM Tris-HCl, pH 8.0), and input samples were de-crosslinked at 55°C for 12 hours in elution buffer (1% SDS and 0.1M NaHCO3) [4]. The DNA was purified with the QIAquick PCR Purification Kit (QIAGEN). qChIPs were performed using PowerSYBRGreen PCRMaster Mix and an ABI PRISM 7500 system (Applied Biosystems). The primer sequences for qChIP are as follows: CD38-F: 5'-CCTTCTGTTGG TGTTGCTGA-3'; CD38-R: 5'-AGGCATGTTGCTTGT GAGTG-3'. CaSR-F: 5'-TCCAGTGTGGTGCTGTGAA T-3'; CaSR-R: 5'-CTTGATGAGGCGGGTAGAGA-3. NFAT-F: 5'-GCTCAGGAGGAGTGAGTGGTA-3'; NF AT-R: 5'-CAGCAGGTTGTGGAGGTTGT-3'.

### NAD^+^ Assay

NAD^+^ levels were measured using a NAD^+^ assay kit (S0175; Beyotime) following the manufacturer’s protocol. The fluorescent product was measured at 540 nm excitation and 620 nm emission wavelengths.

### Cell Viability Assay

Cell viability was assessed using the CCK8 assay. Cells were seeded into 96-well plates and treated with CSE. CCK8 reagent (HY-K0301; MCE) was added, and OD values at 450 nm were measured after 2 hours.

### Histone Extraction

Histone acid extraction from tissues and cells was performed as previously described [31]. The collected tissues or cells were resuspended in lysis buffer containing protease inhibitors (10 mM Tris-HCl, 1 mM KCl, 1.5 mM MgCl_2_, 1 mM DTT, 1.5 mM H_2_SO_4_) and shaken overnight at 4 °C to extract nuclear proteins. The supernatants were then collected after centrifugation at 16,000 × g for 10 minutes at 4 °C, and histones were precipitated on ice using a final concentration of 33% trichloroacetic acid. The histones were washed, dried, and dissolved in H_2_O, and a protein loading buffer was added. Western blotting was performed after incubation for 10 minutes in a metal bath at 98 °C.

### Plasma Membrane Fractionation

Primary AEC2 cells were homogenized in ice-cold homogenization buffer (250 mM sucrose, 10 mM HEPES, pH 7.4, 1 mM EDTA, and protease inhibitors) using a Dounce homogenizer. The homogenate was centrifuged at 1,000 × g for 10 minutes at 4 °C to remove debris and nuclei. The supernatants were then centrifuged at 10,000 × g for 20 minutes to separate the mitochondrial fraction, followed by ultracentrifugation at 100,000 × g for 1 hour to pellet the plasma membrane. The pellet was resuspended and layered onto a discontinuous sucrose gradient (30%, 35%, 40%) and centrifuged at 100,000 × g for 2 hours. The plasma membrane fraction was collected from the 35%-40% interface, washed, and re-pelleted at 100,000 × g for 1 hour. The final pellet was resuspended and stored at -80 °C for further use. Purity was confirmed via western blot using Lamin B1 (nuclear) and GAPDH (membrane and cytosolic) markers.

### Statistical Analysis

Data were analyzed using GraphPad Prism software (version 10.0). All data were expressed as means ± standard deviation (SD), unless otherwise stated. The Shapiro-Wilk normality test was performed to assess the normality of the distribution of data. Due to the small sample sizes, we acknowledge that the assessment of normality may not be reliable. To calculate the comparisons between two groups, unpaired two-tailed Student t tests were performed for normally distributed data (n>5), and Mann-Whitney U tests were performed for nonnormally distributed data or normally distributed data (n≤5). To calculate the comparisons between multiple groups (≥3 groups), one-way analysis of variance (ANOVA) followed by a Bonferroni post hoc test was performed for normally distributed data (n>5), and the Kruskal-Wallis test followed by the Dunn post hoc test was performed for nonnormally distributed data or normally distributed data (n≤5). Welch's t-test and Welch's ANOVA are applied when the data passes the Shapiro-Wilk normality test but has unequal variances (n>5). The number of replicates and other statistical details are reported in the figure legends. *P* < 0.05 was considered statistically significant.

**Figure 1.**
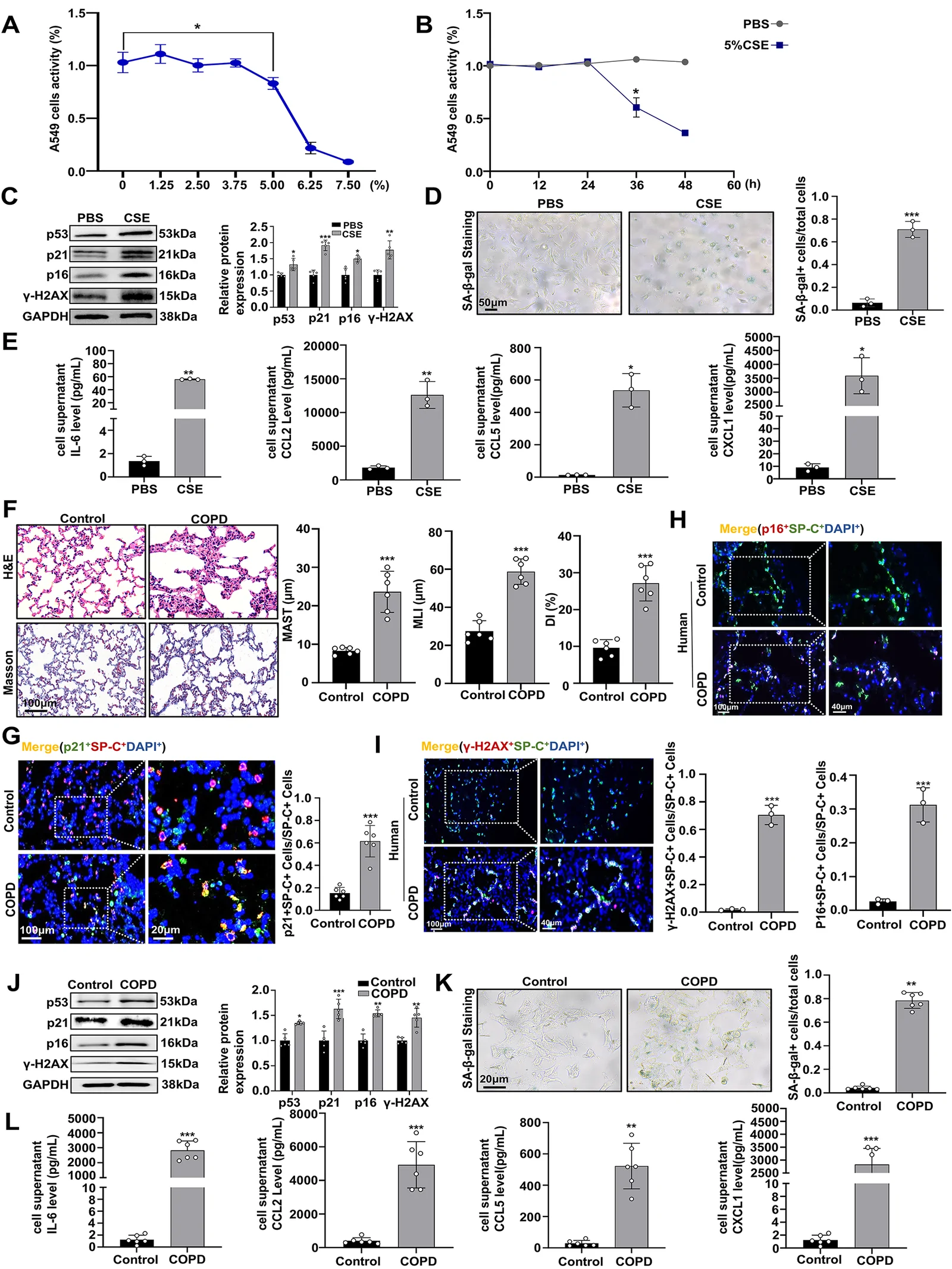
**CSE exposure induces AEC2 senescence**. (**A**) CCK8 assay demonstrating the effect of 1.25% to 7.50% cigarette smoke extract (CSE) stimulation on the viability of the human type II alveolar epithelial cell (AEC2) line A549 after 36 hours (n = 5 per group). (**B**) Viability of A549 cells treated with PBS or 5% CSE over a time course of 0-48 hours (n = 5 per group). (**C**) Western blot analysis of p53, p21, p16, and γ-H2AX protein levels in A549 cells treated with PBS or 5% CSE for 36 hours, relative to GAPDH, with quantification of protein levels shown in the right panel (n = 5 per group). (**D**) SA-β-gal staining images of A549 cells treated with PBS or 5% CSE for 36 hours. Quantification of SA-β-gal positive cells is shown in the right panel (n = 3 per group). (**E**) Quantification of SASP-related cytokines (IL-6, CCL2, CCL5, and CXCL1) levels in supernatants of A549 cells treated with PBS or 5% CSE for 36 hours, assessed by ELISA (n = 3 per group). (**F**) Comparison of lung histological examination (H&E and Masson staining) between the Control and COPD group mice (n = 6 per group). (**G**) Representative immunofluorescence images of p21 (green) co-stained with the AEC2 marker SP-C (red) and nuclear marker DAPI (blue) in mouse lung tissues from Control and COPD mice. Quantification of p21 intensity is shown in the right panel (n =6 per group). (**H**) Representative immunofluorescence images of p16 (red) co-stained with the AEC2 marker SP-C (green) and nuclear marker DAPI (blue) in lung tissues from healthy individuals and patients with COPD. Quantification of p16 intensity is shown in the lower panel (n = 3 per group). (**I**) Representative immunofluorescence images of γ-H2AX (red) co-stained with the AEC2 marker SP-C (green) and nuclear marker DAPI (blue) in lung tissues from healthy individuals and patients with COPD. Quantification of γ-H2AX intensity is shown in the right panel (n = 3 per group). (**J**) Western blot analysis of p53, p21, p16, and γ-H2AX protein levels in primary AEC2s isolated from lung tissues of Control and COPD mice, relative to GAPDH (n = 5 per group), with quantification shown in the right panel. (**K**) SA-β-gal staining images of primary AEC2 from Control and COPD mice (n = 6 per group). Quantification of SA-β-gal-positive cells is shown in the right panel. (**L**) Quantification of SASP-related cytokines (IL-6, CCL2, CCL5, and CXCL1) levels in supernatants of primary AEC2 from Control and COPD group mice, assessed by ELISA (n = 6 per group). The data are represented as the means ± SD of three independent experiments. Two-sided Student’s t-test (F, K), Welch’s t-test (G, L), and Mann-Whitney U tests (A, B, C, D, E, H, I, J) were applied. A-E: *p < 0.05, **p < 0.01, ***p < 0.001, compared with the PBS group. F-L: *p < 0.05, **p < 0.01, ***p < 0.001, compared with the Control group.

## RESULTS

### CSE Exposure Leads to AEC2 Senescence in COPD *In Vitro* and *In Vivo*

To explore the role of AEC2 senescence in COPD, we first established an *in vitro* COPD model by stimulating the AEC2 cell line A549 with varying concentrations of CSE. The CCK-8 assay indicated that the viability of A549 cells was not significantly affected by treatment with 1.25% or 3.75% CSE (Fig. 1A). However, exposure to 5% CSE for 36 hours resulted in a significant reduction in cell viability (Fig. 1B). Based on these results, we selected 5% CSE stimulation for 36 hours as the optimal condition for *in vitro* modeling. Cellular senescence is typically characterized by the upregulation of markers such as p53, p21, p16, and γ-H2AX, increased senescence-associated β-galactosidase (SA-β-gal) activity, and elevated secretion of senescence-associated secretory phenotype (SASP) factors, including IL-6, CCL2, CCL5, and CXCL1. We measured these markers in cells treated with CSE (CSE) and observed a significant increase compared with cells treated with PBS (PBS), consistent with previous studies (Fig. 1C-E). Specifically, the expression of p53, p21, p16, and γ-H2AX increased significantly (Fig. 1C), the proportion of SA-β-gal-positive cells was elevated (Fig. 1D), and levels of SASP cytokines (IL-6, CCL2, CCL5, and CXCL1) in cell supernatants were significantly increased (Fig. 1E). We then constructed a COPD mouse model through 12 weeks of CS exposure. Histological examination of lung tissues using H&E and Masson staining revealed enlarged alveolar spaces and destroyed alveolar walls in COPD mice compared with controls (Fig. 1F). Quantitatively, the mean linear intercept (MLI), destructive index (DI) and mean alveolar septal thickness (MAST) were significantly elevated in COPD mice (Fig. 1F). Furthermore, immunofluorescence co-staining for p21, the AEC2 marker surfactant protein C (SP-C), and the nuclear marker DAPI showed a marked increase in p21 fluorescence in AEC2s of COPD mice compared with control mice (Fig. 1G). Consistently, primary AEC2s isolated from COPD mice exhibited elevated p53, p21, p16, and γ-H2AX levels, an increased proportion of SA-β-gal positive cells, and higher levels of SASP-related cytokines (IL-6, CCL2, CCL5, and CXCL1) in cell supernatants (Fig. 1J-L). In line with these results, immunofluorescence co-staining for p16, γ-H2AX, SP-C, and DAPI also revealed that p16 and γ-H2AX were significantly enhanced in AEC2s from COPD patients compared with healthy controls (Figure 1H, 1I). These results indicate that CSE exposure induces AEC2 senescence both *in vitro* and *in vivo*, suggesting that AEC2 senescence may play a critical role in COPD development and progression.

### CSE Promotes AEC2 Senescence via Lactate-Mediated Histone Lactylation

Previous research has demonstrated that lactate concentrations are heightened in COPD and significantly contribute to disease progression, partly through the activation of the NF-κB signaling pathway [32, 33]. Given that lactate can serve as a substrate for histone lactylation [34], we hypothesized that lactate-mediated histone lactylation is intricately involved in the AEC2 senescence in the context of COPD. To test this hypothesis, we performed western blot analysis of acid-extracted histones and found that Pan-lysine lactylation (Pan Kla) and H4 lysine 12 lactylation (H4K12la) levels increased in lung tissues and isolated primary AEC2s from 12-week-old COPD mice compared to age-matched controls (Fig. 2A). Immunofluorescence co-staining using antibodies against H4K12la (green), AEC2 marker SP-C (red), and nuclear marker DAPI (blue) further confirmed a marked increase in H4K12la fluorescence in AEC2s of COPD mice (Fig. 2B). Consistent with these findings in mice, lung tissue samples from patients with COPD and healthy donors were analyzed. The samples from patients with COPD exhibited a significant increase in H4K12la signals in AEC2s compared to healthy controls (Fig. 2C), supporting the clinical relevance of our findings. Having confirmed the accumulation of H4K12la in both mouse and human COPD lungs, we next sought to determine whether this modification is driven by lactate production *in vitro*.

**Figure 2.**
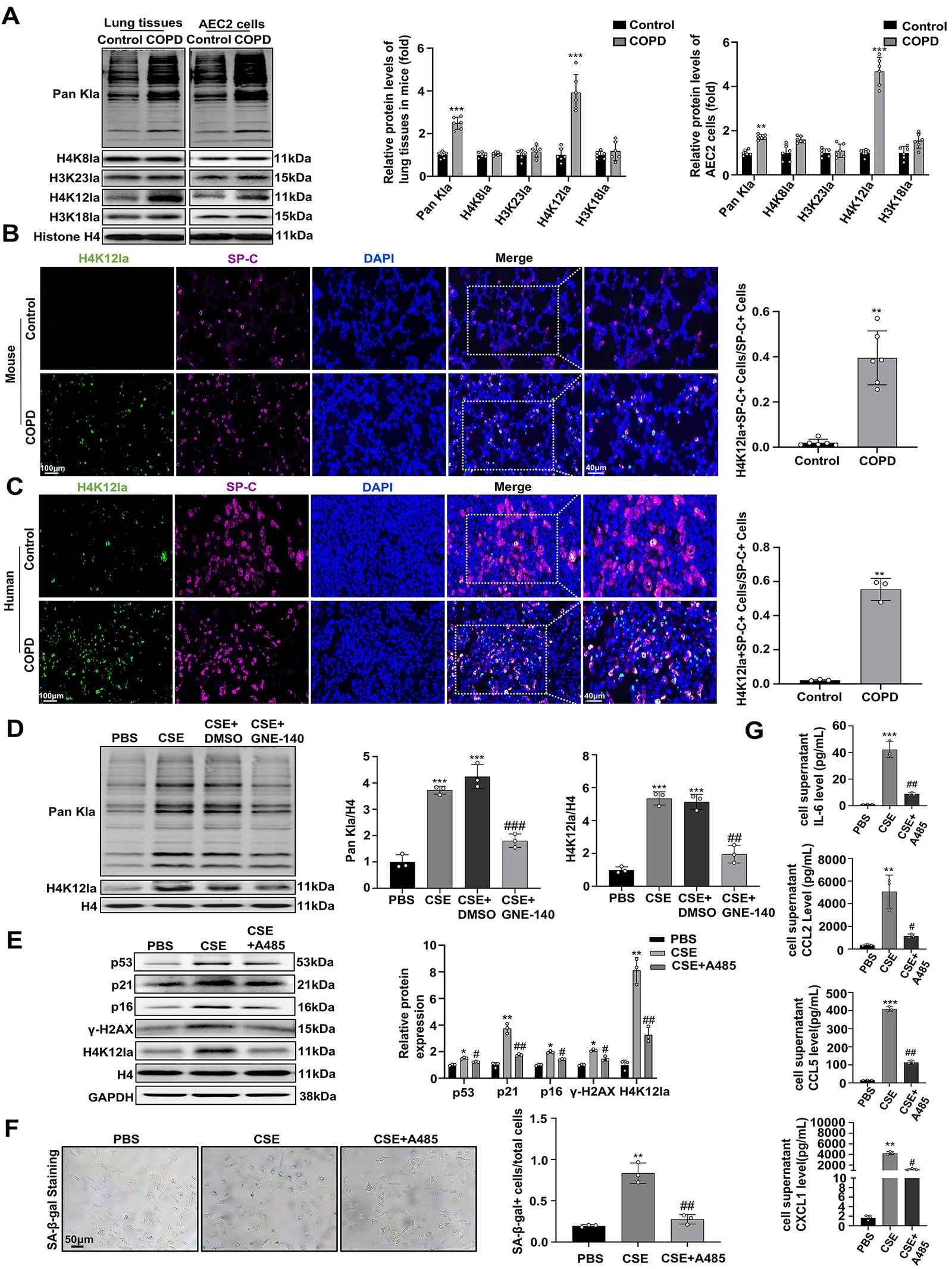
**Elevated lactate levels promote AEC2 senescence through modulation of H4K12la lactylation**. (**A**) Western blot analysis of Pan-Kla and site-specific histone lactylation levels in lung tissues and primary AEC2s isolated from lung tissues of Control and COPD mice, relative to histone H4 (n = 6 per group), as determined by western blot. Quantification of protein levels is shown in the right panel. (**B**) Representative immunofluorescence images of H4K12la (green) co-stained with the AEC2 marker SP-C (red) and nuclear marker DAPI (blue) in lung tissues from Control and COPD mice. Quantification is shown in the right panel (n = 6 mice). (**C**) Representative immunofluorescence images of H4K12la (green) co-stained with the AEC2 marker SP-C (red) and nuclear marker DAPI (blue) in lung tissues from healthy individuals and patients with COPD. Quantification is shown in the right panel (n = 3 per group). (**D**) Western blot analysis showing levels of Pan Kla and H4K12la in A549 cells treated with PBS, CSE, CSE + DMSO, or CSE +GNE-140, relative to histone H4 (n = 3 per group). Quantification is shown in the right panel. (**E**) Western blot analysis of p53, p21, p16, γ-H2AX, and H4K12la levels in A549 cells treated with PBS, CSE, and CSE+A548, the levels of p53, p21, p16, and γ-H2AX were measured relative to GAPDH; the level of H4K12la was measured relative to Histone H4 (n = 3 per group). Quantification is shown in the right panel. (**F**) Representative SA-β-gal staining images of A549 cells under different treatment conditions: PBS, CSE, CSE+A548 (n = 3 per group). Quantification is shown in the right panel. (**G**) Quantification of SASP-related cytokines (IL-6, CCL2, CCL5, and CXCL1) levels in supernatants of A549 cells treated with PBS, CSE, and CSE+A548 (n = 3 per group) on the right. The data are presented as the means ± SD of three independent experiments. Two-sided Student’s t-test (A), Mann-Whitney U tests (B, C), Kruskal-Wallis test followed by the Dunn post hoc test (D, E, F, G) were applied. A-C: **p < 0.01, ***p < 0.001, compared with the Control group. D-G: *p < 0.05, **p < 0.01, ***p < 0.001, compared with the PBS group. #*p* < 0.05, ##*p* < 0.01, ###*p* < 0.001, compared with the CSE group.

To investigate the impact of lactate on H4K12la levels in AEC2 cells under CSE treatment, we treated A549 cells with 5% CSE in the presence or absence of 10 μM GNE-140, a specific inhibitor of lactate dehydrogenase A (LDHA), for 24 hours. By inhibiting LDHA, GNE-140 effectively reduces lactate production within cells. Treatment with GNE-140 significantly attenuated the CSE-induced upregulation of Pan Kla and H4K12la expression (Fig. 2D), indicating that lactate production is necessary for CSE-induced histone lactylation.

H4K12la is an epigenetic modification involving the addition of lactyl groups to the lysine 12 residue of histone H4. Unlike genetic alterations, this modification cannot be directly targeted through gene knockout approaches [35]. Lacylation, a post-translational modification induced by lactic acid, plays an important role in the regulation of gene expression. The mechanism by which lactic acid regulates gene expression via lactylation comprises four key components: the production of lactic acid and its binding to coenzyme A to form lactyl-CoA; the incorporation of the modification by “Writer” proteins; the recognition of lactylation by “Reader” proteins; and its removal by “Eraser” proteins. The key enzymes regulating histone lactylation remain under active investigation [36]. Among known lactyltransferases, p300/CBP is the most well-characterized and has been consistently demonstrated to mediate H4K12la in various biological contexts [37-39]. To investigate the functional role of H4K12la in AEC2 senescence and COPD pathogenesis, we aimed to reduce H4K12la levels by inhibiting p300/CBP, the primary enzyme responsible for this modification. We employed A485, a selective p300/CBP inhibitor with superior efficacy and specificity compared to other inhibitors [26]. A549 cells were treated with 10 μM A485 for 48 hours following cigarette smoke extract (CSE) stimulation. The treatment resulted in reduced expression of H4K12la, along with decreased levels of senescence-associated markers p53, p21, p16, and γ-H2AX (Fig. 2E), a lower proportion of SA-β-gal-positive cells (Fig. 2F), and diminished secretion of SASP-related cytokines (IL-6, CCL2, CCL5, and CXCL1) in cell supernatants (Fig. 2G). These results suggest that CSE promotes AEC2 senescence through a mechanism involving lactate-induced H4K12 lactylation.

### Location of CD38 as Downstream of H4K12la in AEC2s

Histone modifications have the capacity to regulate gene transcription [40], prompting us to investigate the target genes modulated by H4K12la in COPD. CUT&Tag offers high-resolution mapping of histone modifications with reduced background compared to ChIP-seq, enabling precise identification of H4K12la targets. We performed genome-wide CUT&Tag analysis on AEC2s to identify candidate genes regulated by H4K12la [33]. Antibodies against H4K12la were used to enrich for lactylated histones, and libraries were sequenced to an average depth of 30 million reads per sample. Raw sequencing data were processed using deepTools, and we observed a significant enrichment of H4K12la peaks in primary AEC2s from COPD mice compared to controls (Fig. 3A). The comparison revealed 13,277 differential H4K12la binding peaks in primary AEC2s from COPD mice, with 21.11% located within promoter regions (≤3 kb upstream of transcription start sites). In contrast, control samples exhibited only 5,418 differential H4K12la binding peaks, with 17.78% located in promoter regions (Fig. 3B).

**Figure 3.**
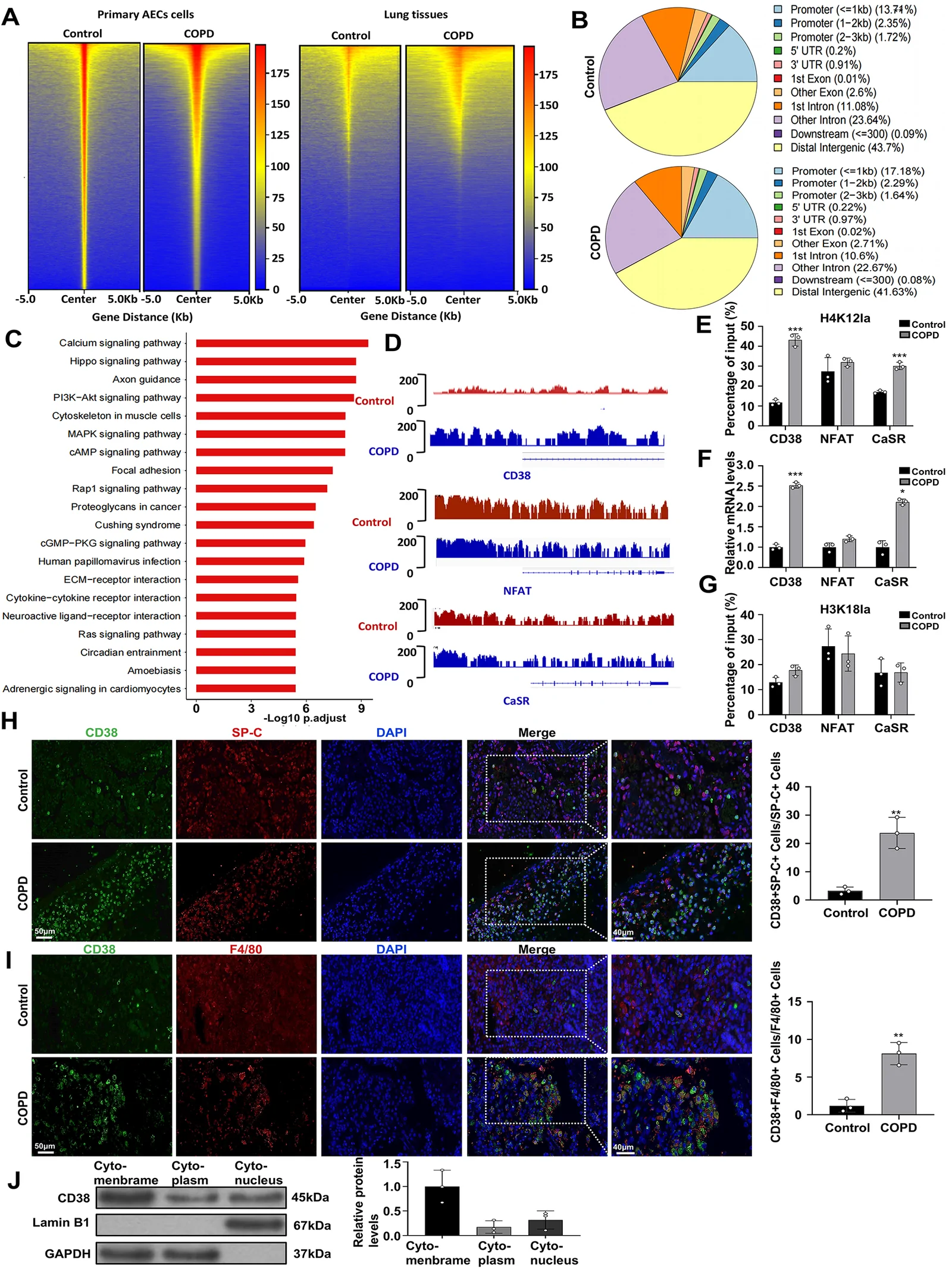
**AEC2 senescence enhances CD38 transcription via histone lactylation**. (**A**) The binding density of H4K12la was visualized using deepTools. The heatmap presents CUT&Tag counts for different H4K12la binding peaks in primary AEC2s (or lung tissue extracts) from Control and COPD mice (n = 5 per group). (**B**) Genome-wide distribution of upregulated H4K12la-binding peaks in primary AEC2s or lung tissues from COPD mice. (**C**) KEGG pathway enrichment analysis of genes associated with elevated H4K12la binding peaks at candidate target loci in primary AEC2s from COPD mice. (**D**) Genome browser tracks displaying CUT&Tag signal at representative target gene loci. Red rectangles highlight peak regions of H4K12la on target gene promoters. (**E**) qChIP analysis of the indicated promoters using H4K12la antibodies in primary AEC2s from Control and COPD mice (n = 3 per group). (**F**) qPCR analysis monitoring expression of the indicated genes in primary AEC2s from Control and COPD mice (n = 3 per group). (**G**) qChIP analysis of the indicated promoters using H3K18la antibodies in primary AEC2s from Control and COPD mice (n = 3 per group). (**H**) Immunofluorescence detection of CD38 (green) in lung tissues from healthy individuals and patients with COPD, co-stained with the AEC2 marker SP-C (red) and nuclei (DAPI, blue) (n = 3 per group). Quantification of CD38 intensity is shown in the right panel. (**I**) Immunofluorescence detection of CD38 (green) in lung tissues from healthy individuals and patients with COPD, co-stained with the macrophage marker F4/80 (red) and nuclei (DAPI, blue) (n = 3 per group). Quantification of CD38 intensity is shown in the right panel. (**J**) Western blot analysis of CD38 levels in the cytomembrane, cytoplasm, and nucleus of A549 cells. CD38 in the cytomembrane and cytoplasm was relative to GAPDH; CD38 in the nucleus was relative to Lamin B1 (n=3 per group). Quantification is shown in the right panel. The data are represented as the means ± SD of three independent experiments. Mann-Whitney U tests (E, F, G, H, I) were applied. E-I: * *p* < 0.05,**p < 0.01, ***p < 0.001, compared with the Control group.

To explore the epigenetic impact of H4K12la in primary AEC2 cells from COPD mice, we classified 4,801 target genes associated with differential H4K12la peaks at promoters into various Kyoto Encyclopedia of Genes and Genomes (KEGG) pathways (Fig. 3C). Notably, the calcium signaling pathway was significantly enriched (Fig. 3C), which is known to regulate cellular processes such as proliferation, apoptosis, and senescence. Other enriched pathways included the Hippo signaling, axon guidance, and the PI3K-Akt signaling pathways (Fig. 3C), all of which are involved in cell proliferation, metabolism, and apoptosis, and have been associated with lung tissue repair and regeneration. Notably, peaks at genomic loci of calcium signaling genes, such as CD38, CaSR, and nuclear factor of activated T-cells (NFAT), showed elevated H4K12la levels at their promoters (Fig. 3D). Given the significant enrichment of H4K12la at these promoters, we sought to validate whether this epigenetic modification leads to increased gene expression. Quantitative chromatin immunoprecipitation (qChIP) assays confirmed increased H4K12la levels at the CD38 and CaSR promoters in primary AEC2s from COPD mice (Fig. 3E), and qPCR analysis validated the elevated expression of CD38 and CaSR in these cells compared with those in AEC2s from control mice (Fig. 3F). Importantly, no significant changes in H3K18la levels at these promoters were observed between COPD and control samples (Fig. 3G), indicating the specificity of H4K12la modifications.

CD38 is a transmembrane protein that regulates calcium signaling by catalyzing the production of cyclic ADP-ribose and degrading NAD^+^, a molecule crucial for cellular metabolism, DNA repair, and immune function [22]. Previous studies have reported elevated CD38 expression in patients with COPD, primarily localized in macrophages, where it contributes to the inflammatory response [41]. However, the role of CD38 in AEC2s has not been thoroughly investigated. Our study reveals a significant increase in CD38 expression not only in macrophages but also in AEC2s of COPD mice (Fig. 3H-I). As shown in Fig. 3H, CD38 expression (green fluorescence) co-localized with SP-C (red fluorescence), which represents AEC2s in lung tissues from COPD mice. Similarly, CD38 expression (green fluorescence) co-localized with F4/80 (red fluorescence), which represents macrophages, increased significantly in lung tissues from COPD mice (Fig. 3I). This novel finding suggests that CD38 upregulation in AEC2s may contribute to COPD progression through mechanisms distinct from those in macrophages. To further explore the subcellular localization of CD38, we performed nuclear membrane fractionation using differential centrifugation to separate membrane, cytoplasmic, and nuclear components (see Methods for detailed protocol). Western blot analysis indicated that CD38 was predominantly localized in the membrane fraction, with lower levels detected in the nuclear and cytoplasmic fractions (Fig. 3J). This membrane localization consists of the role of CD38 as a surface enzyme involved in extracellular metabolism and signaling. Collectively, these findings highlight the upregulation of CD38 in AEC2s and its potential role in cellular senescence of COPD.

### CD38 Promotes AEC2 Senescence Via NAD^+^ Consumption

To assess the functional impact of CD38 on NAD^+^ metabolism, we performed a knockdown of CD38 in A549 cells. The NAD^+^/NADH ratio, measured using a colorimetric assay, was significantly reduced in CSE-treated cells but was partially restored upon CD38 knockdown (Fig. 4A). Simultaneously, under CSE stimulation, knockdown of CD38 partially reversed p53, p21, p16, and γ-H2AX expression, reduced the proportion of SA-β-gal positive cells, and decreased levels of SASP-related cytokines (IL-6, CCL2, CCL5, and CXCL1) in cell supernatants (Fig. 4B-D). These findings suggest that H4K12la promotes AEC2 senescence through CD38 upregulation, contributing to COPD pathogenesis.

**Figure 4.**
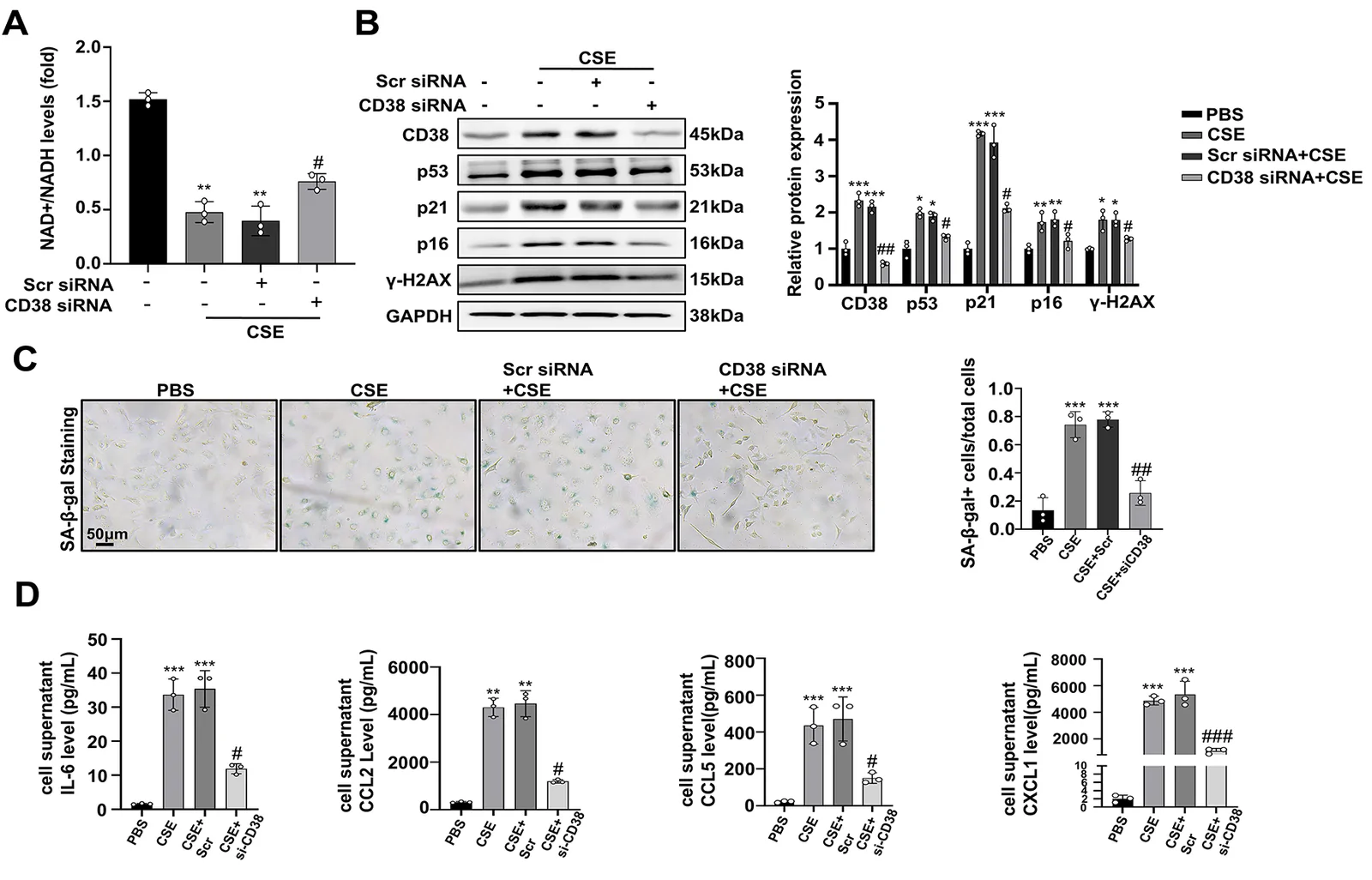
**CD38 Promotes AEC2 Senescence Via NAD+ Consumption**. (**A**) Quantification of NAD^+^/NADH levels in A549 cells under different treatment conditions: PBS, CSE, CSE + Scramble siRNA, and CSE + CD38 siRNA (n = 3 per group). (**B**) Western blot analysis of CD38, p53, p21, p16, and γ-H2AX levels in A549 cells under different treatment conditions: PBS, CSE, CSE + Scramble siRNA, and CSE + CD38 siRNA, relative to GAPDH (n = 3 per group). Quantification is shown in the right panel. (**C**) SA-β-gal staining images of A549 cells under different treatment conditions: PBS, CSE, CSE + Scramble siRNA, and CSE + CD38 siRNA, with quantification of SA-β-gal positive cells (n = 3 per group). Quantification is shown in the right panel. (**D**) Quantification of SASP-related cytokine levels (IL-6, CCL2, CCL5, and CXCL1) in supernatants of A549 cells under different treatment conditions: PBS, CSE, CSE + Scramble siRNA, and CSE + CD38 siRNA (n = 3 per group). The data are represented as the means ± SD of three independent experiments. Kruskal-Wallis test followed by the Dunn post hoc test (A, B, C, D) was applied. *p < 0.05, **p < 0.01, ***p < 0.001, compared with the PBS group. #*p*< 0.05, ##*p*< 0.01, ###*p* < 0.001, compared with the CSE + Scramble siRNA group.

### The Small Molecule Inhibitor A485 of p300/CBP Alleviates AEC2 Senescence and COPD Pathology in COPD Model Mice Through H4K12la Modification

Based on prior studies demonstrating the efficacy and safety of A485 at a dosage of 25 mg/kg/day in mouse models [26], we administered A485, a selective inhibitor of p300/CBP, via oral gavage once daily for 4 weeks. Oral gavage was chosen to ensure precise dosing and consistent systemic exposure. *In vivo* experiments demonstrated that A485 significantly decreased H4K12la fluorescence intensity (green) co-localizing with SP-C (red) compared to untreated COPD mice by immunofluorescence co-staining (Fig. 5A). Western blot analysis showed A485 treatment significantly reduced H4K12la levels in AEC2s of COPD mice (Fig. 5B). A485 treatment also led to decreased expression levels of CD38, p53, p21, p16, and γ-H2AX (Fig. 5C), reduced SA-β-gal positivity (Fig. 5D), and lowered levels of SASP-related cytokines (IL-6, CCL2, CCL5, and CXCL1) in cell supernatants (Fig. 5E). Concurrently, A485 treatment elevated the NAD^+^/NADH ratio in primary AEC2s of COPD mice (Fig. 5F). Histopathological evaluations indicated that A485-treated mice exhibited reduced alveolar destruction and fibrosis compared to untreated COPD mice (Fig. 5G). Lung function tests were conducted, revealing significant improvements in FEV1/FVC ratios among mice treated with A485 compared to those with COPD without any treatment (Fig. 5H). These findings indicate that A485 ameliorated COPD-associated lung damage and improved respiratory function.

Collectively, these findings suggest that A485 mitigates COPD pathology by reducing cellular senescence and restoring NAD^+^ homeostasis through the inhibition of H4K12la modification.

**Figure 5.**
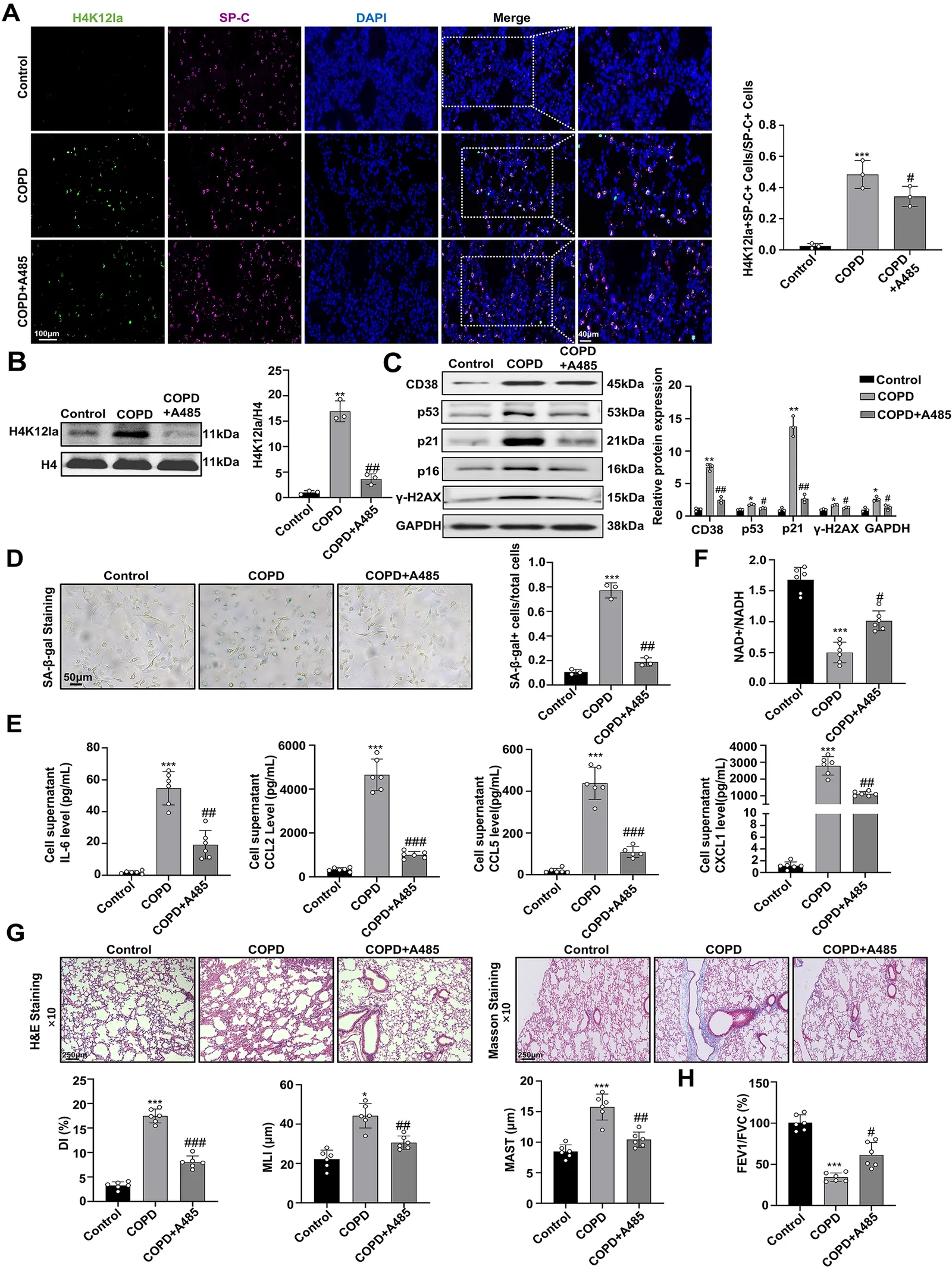
**A485, a small molecule inhibitor of H4K12la, ameliorates AEC2 senescence and pathology in COPD mice**. (**A**) Representative immunofluorescence images of H4K12la co-stained with the AEC2 marker SP-C (red) and nuclei (DAPI, blue) in lung tissues from Control, COPD, and COPD + A485-treated mice (n = 3 per group). Quantification of CD38 intensity is shown in the right panel. (**B-C**) Western blot analysis of H4K12la, CD38, p53, p21, p16, and γ-H2AX protein levels in primary AEC2s from Control, COPD, and COPD + A485-treated mice (n = 3 per group), the level of H4K12la was measured relative to Histone H4, and the levels of CD38, p53, p21, p16, and γ-H2AX were measured relative to GAPDH. Quantification is shown in the right panel. (**D**) SA-β-gal staining images of primary AEC2s from Control, COPD, and COPD + A485-treated mice (n = 3 per group). Quantification is shown in the right panel. (**E**) Quantification of SASP-related cytokines (IL-6, CCL2, CCL5, and CXCL1) in supernatants of primary AEC2s from Control, COPD, and COPD + A485-treated mice, as determined by ELISA (n = 6 per group). (**F**) Comparison of the NAD+/NADH ratio in primary AEC2s from Control, COPD, and COPD + A485-treated mice (n = 6 per group). (**G**) Comparison of lung histological examination by H&E and Masson staining images of lung tissues from Control, COPD, and COPD + A485-treated mice, with quantification presented in the lower panel (n = 6 per group). (**H**) Comparison of lung function test results in Control, COPD, and COPD + A485-treated mice (n = 6 per group). The data are represented as the means ± SD of three independent experiments. Kruskal-Wallis test followed by the Dunn post hoc test (A, B, C, D), one-way analysis of variance (ANOVA) followed by Bonferroni post hoc test (E, F, G, H) were applied. *p < 0.05, **p < 0.01,***p < 0.001, compared with the Control group. *#p* < 0.05, ##*p* < 0.01, ###*p* < 0.001, compared with the COPD group.

### CD38 Inhibition or NAD^+^ Supplementation Alleviates CSE/H4K12la/CD38-Induced AEC2 Senescence

CD38 can be categorized into either type II or type III based on its orientation across the cell membrane. In the more prevalent type II configuration, the catalytic domain of CD38 is oriented toward the extracellular space, where molecules such as NAD^+^, nicotinamide mononucleotide (NMN), ADPR, cADPR, and their downstream metabolites-adenosine monophosphate (AMP) and adenosine - are predominantly found. In contrast, for the type III isoform of CD38 localized on the plasma membrane, the catalytic site faces the cytoplasmic side, enabling it to directly regulate intracellular NAD+ levels and cADPR production [42, 43]. These findings suggest that CD38 may perform distinct functional roles through different structural conformations. 78c, as a specific inhibitor of CD38, reverses the decline in NAD levels and inhibits age-related cellular senescence by targeting the full-length CD38 molecule [44, 45]. To investigate the role of CD38 in AEC2 cell senescence under CSE stimulation, we pretreated A549 cells with 1.0 μM 78c, 100 μM nicotinamide mononucleotide (NMN, an NAD^+^ precursor), or their combination for 4 hours before CSE exposure. These concentrations were selected based on previous studies demonstrating efficacy in modulating CD38 activity and enhancing NAD^+^ levels [22]. Western blot analysis showed that 78c, NMN, and their combination (78c + NMN) significantly reduced p53, p21, p16 and γ-H2AX protein levels (Fig. 6A), decreased SA-β-gal positive cells (Fig. 6B), downregulated SASP-related cytokines (IL-6, CCL2, CCL5, and CXCL1) in cell supernatants (Fig. 6C), and increased the NAD^+^/NADH ratio (Fig. 6D).

*In vivo*, we administered 78c (15 mg/kg/day), NMN (300 mg/kg/day), or their combination (78c + NMN) to COPD mice via oral gavage once daily for 4 weeks, starting at the 8th week after establishing the COPD model, following protocols from prior research. Western blot analysis of lung tissues showed that either 78c or NMN treatments individually, or their combination treatment reduced p53, p21, p16 and γ-H2AX protein expression levels compared to untreated COPD mice (Fig. 6E). SA-β-gal staining of lung sections revealed a significant decrease in senescent AEC2s (Fig. 6F). All of the treatments led to lower levels of SASP-related cytokines (IL-6, CCL2, CCL5, and CXCL1) in cell supernatants (Fig. 6G) and significantly elevated NAD^+^/NADH levels in lung tissues (Fig. 6H), indicating restoration of NAD^+^ homeostasis. Histopathological examinations showed reduced alveolar destruction, inflammation, and fibrosis in treated mice (Fig. 6I). Lung function tests demonstrated improvements in FEV1/FVC ratios compared to untreated COPD mice (Fig. 6J), indicating that targeting the CD38-NAD^+^ axis, whether through 78c, NMN, or their combination, can mitigate AEC2 senescence and slow COPD progression.

Interestingly, the combined treatment of 78c and NMN did not provide additional benefits in reducing cellular senescence or improving lung pathology compared to the effects of 78c or NMN alone. This lack of synergy may be due to a saturation effect on the CD38-NAD^+^ pathway, where maximal restoration of NAD^+^ levels and reduction of senescence markers are achieved with individual treatments. Alternatively, compensatory mechanisms or feedback inhibition within the NAD^+^ salvage pathway may limit the combined efficacy.

### Mechanism of H4K12la Induces AEC2 Senescence in COPD by Regulating the CD38-NAD^+^ pathway

During the progression of COPD, increased lactate levels in AEC2s lead to the lactylation of H4K12la. This modification acts as a key trigger for cellular senescence in AEC2s, contributing to disease progression. The study illustrates how inhibiting H4K12la and its downstream signaling pathway, specifically the CD38-NAD^+^ axis, can mitigate AEC2 senescence and reduce inflammatory responses. Experimental interventions targeting H4K12la showed a reduction in these pathological effects, emphasizing the potential of modulating the lactylation network to manage COPD effectively (Fig. 7).

**Figure 6.**
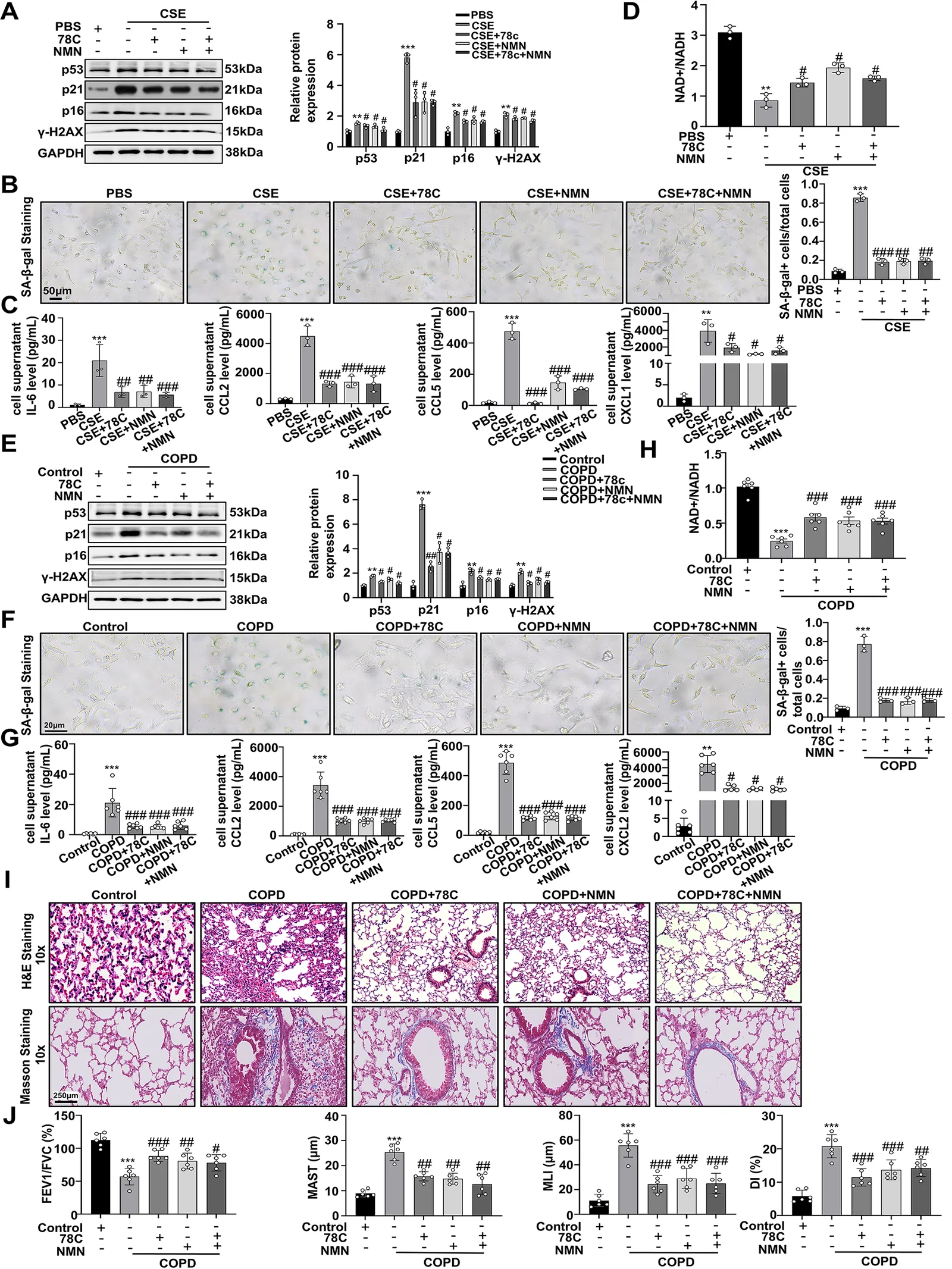
**Exogenous administration of 78c and/or NMN mitigates AEC2 senescence in COPD mice by improving NAD^+^ levels**. (**A**) Western blot analysis of p53, p21, p16, and γ-H2AX levels in A549 cells under different treatment conditions: PBS, CSE, CSE +78c, CSE+NMN, and CSE+78c+NMN (n = 3 per group), relative to GAPDH. Quantification is shown in the right panel. (**B**) SA-β-gal staining images of A549 cells under different treatment conditions: PBS, CSE, CSE+78c, CSE+NMN, and CSE+78c+NMN (n = 3 per group). Quantification is shown in the right panel. (**C**) Quantification of SASP-related cytokines (IL-6, CCL2, CCL5, and CXCL1) levels in supernatants of A549 cells under different treatment conditions: PBS, CSE, CSE +78, CSE+NMN, and CSE+78c+NMN (n = 3 per group). (**D**) Comparison of NAD+/NADH ratio in A549 cells under different treatment conditions: PBS, CSE, CSE + 78c, CSE+NMN, and CSE+78c+NMN (n = 3 per group). (**E**) Western blot analysis of p53, p21, p16, and γ-H2AX levels in primary AEC2s from control and COPD mice under different treatment conditions: PBS, COPD+PBS, COPD+78c, COPD+NMN, and COPD+78c+NMN (n = 3 per group), relative to GAPDH. Quantification is shown in the right panel. (**F**) SA-β-gal staining images of primary AEC2 cells from control and COPD mice under different treatment conditions: PBS, COPD+PBS, COPD+78c, COPD+NMN, and COPD+78c+NMN (n = 3 per group). Quantification is shown in the right panel. (**G**) Quantification of SASP-related cytokines (IL-6, CCL2, CCL5, and CXCL1) levels in supernatants of primary AEC2 cells from control and COPD mice under different treatment conditions: PBS, COPD+PBS, COPD+78c, COPD+NMN, and COPD+78c+NMN (n = 6 per group). (**H**) Comparison of NAD^+^/NADH ratio in primary AEC2s from control and COPD mice under different treatment conditions: PBS, COPD+PBS, COPD+78c, COPD+NMN, and COPD+78c+NMN (n = 6 per group). (**I**) Comparison of lung histological examination by H&E and Masson staining images of lung tissue from control and COPD mice under different treatment conditions: PBS, COPD+PBS, COPD+78c, COPD+NMN, and COPD+78c+NMN (n = 6 per group). Quantification is shown in the lower panel. (**J**) Lung function test results in control and COPD mice under different treatment conditions: PBS, COPD+PBS, COPD+78c, COPD+NMN, and COPD+78c+NMN (n = 6 per group). The data are represented as the means ± SD of three independent experiments. Kruskal-Wallis test followed by the Dunn post hoc test (A, B, C, D, E, F), one-way analysis of variance (ANOVA) followed by Bonferroni post hoc test (G, H, I, J) were applied. A-D: **p < 0.01, ***p < 0.001, compared with the PBS group. #*p* < 0.05, ##*p* < 0.01, ###*p* < 0.001, compared with the CSE group. E-J: **p < 0.01, ***p < 0.001, compared to the Control group. #*p* < 0.05, ##*p* < 0.01, ###*p* < 0.001, compared with the COPD group.

## DISCUSSION

COPD is a progressive lung disease characterized by chronic inflammation, airway remodeling, and alveolar destruction, leading to impaired respiratory function [46]. A critical cellular mechanism in COPD is the senescence of AEC2s, which has emerged as a significant contributor to disease progression [47]. AEC2s play a crucial role in maintaining alveolar homeostasis due to their ability to self-renew and differentiate into alveolar epithelial type I cells (AEC1), which form the alveolar surface and facilitate gas exchange [2]. Chronic exposure to CS and persistent inflammation accelerate AEC2 senescence through oxidative stress-induced DNA damage and activation of the p53/p21 pathway, leading to cell cycle arrest and impaired regenerative capacity [26, 48]. Additionally, inflammatory cytokines can activate signaling pathways further promoting senescence and weakening tissue integrity [48]. Senescent AEC2s may undergo SASP, which creates a sustained inflammatory microenvironment that further exacerbates lung injury and drives disease progression [49]. In our study, we confirmed the presence of senescent AEC2s in COPD mouse models by employing immunohistochemical staining and ELISA to detect elevated levels of senescence markers such as p53, p21, p16, γ-H2AX, β-gal-positive cells, and SASP-related factors. These findings align with existing literature linking AEC2 senescence to COPD pathology, underscoring the therapeutic potential of targeting senescent AEC2s. Potential therapeutic strategies include the development of senolytic agents that selectively eliminate senescent cells [50]. However, challenges associated with senolytic agents, such as toxicity and treatment duration, limit their clinical application in COPD [50]. Therefore, the development of senomorphic drugs or natural products that modulate the AEC senescence-related metabolism to reduce SASP is becoming promising.

Epigenetic modifications are increasingly recognized as critical regulators of cellular aging and chronic disease development [51, 52]. Among these, histone lactylation, particularly at lysine 12 of histone H4 (H4K12la), has emerged as a novel modification influencing gene expression [53, 54]. Histone lactylation involves the addition of lactate to lysine residues on histone proteins, affecting chromatin structure and transcriptional activity [55]. Although lactate has traditionally been regarded as a metabolic byproduct of anaerobic glycolysis, it is now understood to function as a signaling molecule capable of modulating gene expression through histone modifications [56, 57]. In COPD, chronic hypoxia and inflammation induce metabolic reprogramming towards anaerobic glycolysis, leading to increased lactate production [58]. Our study revealed a significant increase in H4K12la levels within AEC2s from both murine and human COPD samples, as evidenced by western blot and immunofluorescence staining (Fig. 2A-C). Furthermore, inhibition of lactate production using the LDHA inhibitor GNE-140 attenuated CSE-induced H4K12la elevation (Fig. 2D), supporting the notion that lactate serves as the substrate for this modification in our model. Genome-wide CUT&Tag profiling in AEC2s identified enriched H4K12la peaks at the promoters of genes involved in critical pathways, notably including CD38 (Fig. 3A-D), which was validated by qChIP-qPCR (Fig. 3E-F). Subsequent functional experiments demonstrated that reducing H4K12la (via A485) or its downstream target CD38 (via siRNA or 78c) ameliorated cellular senescence, thereby establishing a causal link between lactate-driven H4K12la and AEC2 senescence in COPD. This hypothesis is supported by studies in other disease models, such as Alzheimer’s disease and cancer, where histone lactylation promotes inflammatory gene expression and senescence-like phenotypes [54, 59]. Using the LDHA inhibitor GNE-140, we were able to reduce lactate production, leading to decreased H4K12la levels in AEC2s and reduced expression of senescence markers. However, considering essential roles of lactate in normal cellular physiology, including functioning as an energy source and a substrate for gluconeogenesis [60], targeting lactate metabolism may present therapeutic challenges due to potential side effects on normal tissues [61]. Future strategies may focus on selectively inhibiting histone lactylation or targeting specific downstream effects of H4K12la to minimize unintended consequences.

**Figure 7.**
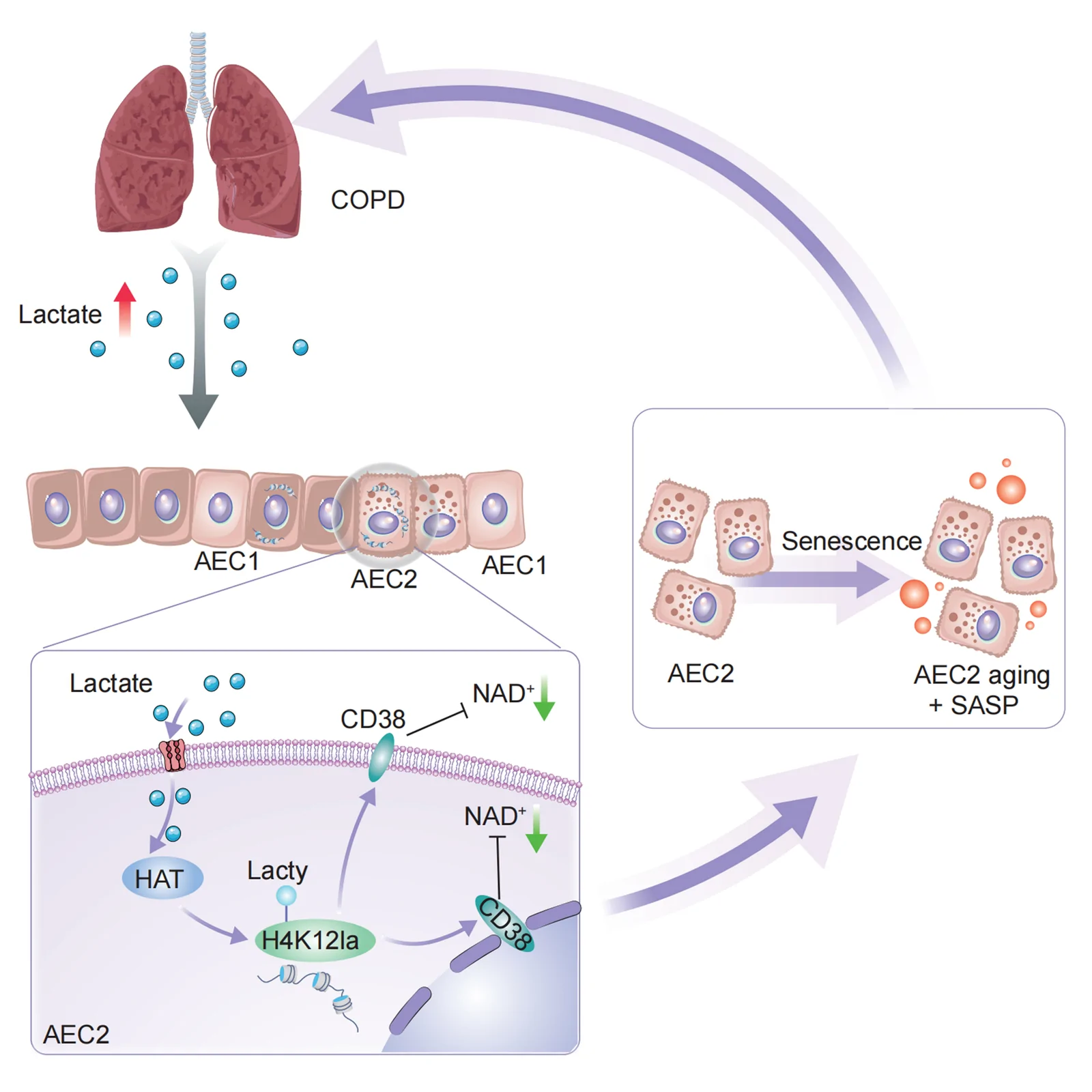
**Mechanism diagram**. This diagram illustrates the mechanism of COPD progression through lactate accumulation and subsequent lactylation modification of histone H4 lysine 12 (H4K12la) in alveolar epithelial type 2 cells (AEC2s). The diagram shows how H4K12la triggers AEC2 senescence, contributing to inflammation and COPD advancement. It also highlights the inhibition pathway involving H4K12la and its downstream signaling cascade, CD38-NAD^+^. The study proposes that targeting this pathway can ameliorate AEC2 senescence and improve COPD outcomes by disrupting the lactylation modification signaling network.

To specifically target histone lactylation, we evaluated A485, a selective inhibitor of p300/CBP. These enzymes possess histone lactyltransferase activity and regulate H4K12la [26]. In COPD mouse models, A485 treatment effectively reduced H4K12la levels, decreased CD38 expression, and alleviated senescence markers such as p53, p21, p16, γ-H2AX, SA-β-gal, and SASP cytokines (IL-6, CCL2, CCL5, and CXCL1) (Fig. 5A-E). Furthermore, A485 restored the NAD^+^/NADH ratio, improved lung pathology, and enhanced respiratory function in COPD mice (Fig. 5F-H), implying its potential to mitigate senescence driven by the H4K12la-CD38-NAD^+^ axis. This finding indicated that A485 is a promising small-molecule inhibitor capable of targeting lactylation-mediated epigenetic regulation in COPD. Although other enzymes, such as HDACs, may also regulate histone lactylation [62], their roles in AEC2 senescence are not yet clear and merit further investigation.

Beyond COPD, H4K12la is implicated in various chronic inflammatory and aging-related diseases [35, 63, 64]. In inflammatory conditions, H4K12la has been linked to the upregulation of pro-inflammatory genes, exacerbating inflammation [65]. In aging-related diseases like osteoarthritis and neurodegeneration, H4K12la has been associated with cellular senescence and tissue degeneration by regulating pathways such as p53, NF-KB, and ROS that regulate cellular stress responses and apoptosis [64, 66]. Nevertheless, most of these findings are based on preclinical models, and clinical validation in human subjects is limited. These findings highlight H4K12la as a common epigenetic mechanism across multiple pathologies associated with chronic inflammation and aging. Our study adds valuable insight to this growing body of evidence by identifying the role of H4K12la in AEC2 senescence in COPD. Future directions may include exploring targeted epigenetic therapies that specifically modulate H4K12la, investigating combinatory treatments that address multiple senescence pathways.

Through CUT&Tag sequencing analysis, we observed that H4K12la is enriched at the promoters of genes involved in several key signaling pathways, including calcium, Hippo, and PI3K-Akt signaling pathways, which regulate cellular stress responses, proliferation, apoptosis, and inflammation. To validate these findings, we performed qChIP assays to confirm the enrichment at these promoters. Among these genes, we identified a pronounced enrichment of H4K12la at the promoter of the CD38 gene, indicating a specific regulatory role for H4K12la in modulating CD38 expression. CD38 is a multifunctional enzyme crucial for NAD^+^ metabolism and calcium signaling, playing a vital role in cellular energy balance, mitochondrial function, and inflammatory responses [16]. By catalyzing the conversion of NAD^+^ to cADPR, CD38 mobilizes calcium from intracellular stores and influences cellular processes, including DNA repair and stress responses [67]. Increased CD38 expression, potentially driven by H4K12la enrichment, leads to NAD^+^ depletion, mitochondrial dysfunction, and cellular senescence - key features of COPD pathogenesis. This finding suggests a direct mechanistic pathway by which H4K12la-induced CD38 upregulation contributes to metabolic dysregulation and cellular aging. Considering that CD38 upregulation has been reported in lung tissues of patients with COPD and correlates with disease severity [19], targeting the H4K12la-CD38 axis presents a promising therapeutic approach. The pivotal role of CD38 in NAD^+^ catabolism establishes it as a central mediator of senescence within the proposed H4K12la-CD38-NAD^+^ axis. While cellular NAD^+^ homeostasis is dynamically regulated by both biosynthetic pathways-such as the NAMPT-mediated salvage pathway, the primary NAD^+^ source in mammals-and consuming enzymes including PARPs, SIRTs, and SARM1 [68,69], our findings highlight CD38 as the NAD^+^ hydrolase in COPD-related AEC2 senescence, driven by H4K12la. Notably, Lai et al. reported reduced NAMPT expression in senescent AEC2s during pulmonary fibrosis, which impairs NAD^+^ regeneration via the salvage pathway [15]. This suggests a potential synergy between CD38 upregulation and NAMPT downregulation in exacerbating NAD^+^depletion. Furthermore, Zhang et al. demonstrated that in cigarette smoke-induced senescence, PARP1 hyperactivation competitively consumes NAD^+^, thereby suppressing SIRT1-dependent autophagy and perpetuating a cycle of DNA damage [70]. Given these insights, targeting the H4K12la-CD38 interaction may offer a more precise therapeutic strategy for restoring NAD^+^ homeostasis and alleviating AEC2 senescence in COPD, as opposed to modulating broader pathways such as Hippo or PI3K-Akt, which may not be directly influenced by H4K12la. Given that NAMPT downregulation impairs NAD^+^ regeneration in senescent cells [15], the lack of synergy we observed between the CD38 inhibitor 78c and the NAD^+^ precursor NMN suggests that merely providing substrate may be insufficient to counteract all mechanisms of NAD^+^ depletion. Future strategies could therefore explore combining CD38 inhibition with agents that directly target the salvage pathway, such as NAMPT activators, to test if a more comprehensive blockade of NAD^+^ consumption coupled with enhanced synthesis can yield greater efficacy.

Our findings validate targeting the H4K12la-CD38-NAD^+^ axis as a viable anti-senescence strategy. We selected A485 - a potent p300/CBP inhibitor - based on compelling evidence that p300/CBP is the primary "writer" for H4K12la [12, 40], and its inhibition effectively suppresses lactylation-driven pathologies, including lung fibrosis [74]. While other enzymes (e.g., HDAC1-3, SIRT1-3) have been reported to act as "erasers" of lactylation in other contexts [75, 76], their specific roles, if any, in AEC2 senescence remain to be investigated. Similarly, for CD38 inhibition, we employed 78c due to its ability to target full-length CD38 - effectively inhibiting both extracellular (type II) and intracellular (type III) enzymatic conformations that collectively deplete NAD^+^ [24, 77]. Pharmacological inhibition of CD38 with 78c or NAD^+^ repletion with NMN similarly mitigated senescence markers (p53, p21, p16, γ-H2AX) and SASP, confirming NAD^+^ restoration as the critical mechanistic endpoint. The lack of synergy between 78c and NMN suggests a saturation effect in NAD^+^ rescue, though dose optimization studies may further refine this approach.

Our study provides compelling evidence that H4K12la functions as a critical epigenetic regulator in AEC2 senescence and COPD progression, primarily through upregulating CD38, which leads to NAD^+^ depletion. By identifying CD38 as a direct target of H4K12la, we establish a novel H4K12la-CD38-NAD^+^ axis linking metabolic stress with cellular aging in COPD. The therapeutic implications of these findings suggest that modulating this axis - through CD38 inhibition or NAD+ precursor supplementation - could offer an effective strategy for mitigating AEC2 senescence and slowing COPD progression. Future studies should explore broader gene regulatory effects of H4K12la and identify potential lactylases or delactylases for more precise epigenetic interventions. Ultimately, this work advances our understanding of COPD and opens new therapeutic possibilities for chronic inflammatory and aging-related diseases.
